# Evolution of Sexual Dimorphism in the Digit Ratio 2D:4D - Relationships with Body Size and Microhabitat Use in Iguanian Lizards

**DOI:** 10.1371/journal.pone.0028465

**Published:** 2011-12-05

**Authors:** Camilla M. Gomes, Tiana Kohlsdorf

**Affiliations:** Department of Biology, Faculdade de Filosofia, Ciências e Letras de Ribeirão Preto, University of São Paulo, Ribeirão Preto, SP, Brazil; Ecole Normale Supérieure de Lyon, France

## Abstract

The ratio between lengths of digit II and IV (digit ratio 2D:4D) is a morphological feature that likely affects tetrapod locomotor performances in different microhabitats. Modifications of this trait may be triggered by changes in steroids concentrations during embryo development, which might reflect direct selection acting on digit ratio or be solely a consequence of hormonal differences related for example to body size. Here we apply both conventional and phylogenetic analyses on morphological data from 25 lizard species of 3 families of Iguania (Iguanidae, Polychrotidae, and Tropiduridae), in order to verify whether selective pressures related to locomotion in different microhabitats could override the prenatal developmental cues imposed on the digit ratio 2D:4D by differences in body size between males and females. Data suggest that this trait evolved in association with ecological divergence in the species studied, despite the clear effect of body size on the digit ratio 2D:4D. The ecological associations of size-corrected digit ratios were restricted to one sex, and females of species that often use perches exhibited small digit ratios in the front limbs, which translated into larger sexual dimorphism indexes of arboreal species. The results, together with the subsequent discussion, provide outlines for further investigation about possible developmental mechanisms related to the evolution of adaptive changes in digit lengths that may have occurred during the evolution of ecological divergence in squamates.

## Introduction

The association between ecological diversity and morphological variation is very well documented in several animal taxa (e.g. Anurans: [1]–[5], Reptiles: [6]–[13], Mammals: [14]–[19]), although information about the mechanisms that probably elicit evolutionary changes in morphology during a process of ecological divergence is still scarce and restricted to few biological groups (for examples see [20], [21]). Evolutionary changes in the shape and size of a given structure are believed to reside on alterations that occurred during embryo development [22]–[27], but the evolution of morphological variation is very often inferred from adult morphologies, probably because in nature embryos are usually hard to access. The relationship between morphological divergence and possible deviations from an ancestral developmental program is difficult to infer also because the new shape or size of a given structure may either be an evolutionary response to direct selection or a consequence of selection acting on a different trait. For example, differences in the selective pressures related to microhabitats that are structurally distinct might result in morphological variation of structures used during locomotion, but such variation could also be a consequence of selection acting on other traits, such as clutch size or body mass. One morphological feature that may exemplify these postulated scenarios is the digit ratio 2D:4D. This trait likely affects locomotion, and therefore may diverge in response to ecological differentiation among lineages, but it is also possible that digit proportions modify exclusively as a consequence of changes in developmental programs triggered by variation in body size.

The ratio between the lengths of digit II and IV in tetrapods (digit ratio 2D:4D; [28]) is determined during embryo development, and differences between males and females may evolve through changes in prenatal steroids concentrations. In some mammals, females have larger digit ratios 2D:4D than males [29]-[31], in contrast with species of reptiles and birds where males have usually higher values than females [e.g. 32–34]. This morphological feature is established mostly during the embryonic period, and changes little after sexual maturation [35]–[39]. Limb development and digit elongation are coordinated by expression of *Hox* genes (for example, *Hoxa-13* [40] and *Hoxd-13* [41]), which in turn is directly affected by the concentration of sex hormones (testosterone and estrogen) in the embryo [42]. In this context, a subtle variation in the embryo's sexual steroids concentrations seems sufficient to modify the relative lengths of digits II and IV observed in adults [36], [37], [43]. The actions of androgens and estrogens on digit development are distinct: estrogen concentration positively affects the length of digit II, while testosterone levels seem related to the length of digit IV [28]. Consequently, the ratio between the lengths of digits II and IV (2D:4D) may be sexually dimorphic, which would be modulated by the differential concentration of these two hormones in developing male and female embryos [44]. It is not clear, however, if these probable differences in hormone levels between sexes are always primarily related to variation in sexual traits, such as body size, or if this developmental program could deviate from the ancestral state by selection acting directly on the digit ratio 2D:4D.

Sexual dimorphism in digit ratio 2D:4D has been reported in almost all tetrapod groups (lizards: [34], [35]; birds: [32]; mammals: [31], [45]; and particularly humans: [28], [42], [46]–[48]), but the historical processes that might lead to evolution of this pattern remain obscure, and it is not clear whether differences between males and females in autopodial morphology could have evolved independently from the divergences in body size. The connection between relative digit lengths and microhabitat usage is very feasible, given that in several taxa foot morphology is associated for example with the locomotor performance exhibited in different ecological settings (e.g. [49]–[56]), and may as well affect other ecological activities, as basking, foraging, and mating. Evolution of autopodium morphology in the context of habitat usage is particularly well investigated in squamates, where evolution of limb and foot lengths seems clearly associated with locomotion on different surfaces (e.g. [9], [11], [13], [53], [57]–[59]). Lizards constitute a great model for the investigation of this relationship because of their extensive morphological variation, great ecological diversity, and broad use of different locomotor modes, such as running, jumping, and climbing (e.g. [60]–[65]). Moreover, associations between morphology and ecology have been reported in several families, as Polychrotidae (particularly *Anolis* [6], [7], [57], [66]–[68]), Iguanidae [69], and Tropiduridae [9], [10], [13], [64], [70]–[72]. Some studies suggest for example that species from sandy environments exhibit longer feet (e.g. [9], [13]), although variation in squamate autopodium morphology in the context of habitat use is often inferred based exclusively on the length of the fourth toe. Thus, investigation of morphological divergence in this structure usually does not consider differences among digit ratios, and to our knowledge possible ecological associations of the digit ratio 2D:4D remain unexplored in a comparative framework. One complicating factor is that variation in this feature may also reflect hormonal changes related to differences in body size, and there is evidence that the snout-vent length is associated with ecological divergence in some lizard groups (e.g. [66], [71]). In this scenario, it remains obscure whether the digit ratio may evolve in association with ecological divergence in the absence of correlations between ecology and body mass. Such disconnection would provide evidence that ecological relationships of digit proportions may override the developmental program imposed exclusively by variation in steroids concentrations due to size differences between males and females.

Based on the theoretical context presented here, it is conceivable that the digit ratio 2D:4D may have evolved in parallel with ecological diversity in some clades of Squamata. even if part of the differences between sexes observed in this trait likely reflects variation in hormone levels related to divergence in body size. For example, it is plausible to expect that arboreal species would exhibit larger differences among digit lengths in the autopodium (i.e. smaller digit ratios), which would be particularly relevant for lizards that occupy the canopies and face a three-dimensional structural complexity composed by perches disposed on various orientations. However, if most of the arboreal species also exhibit larger body sizes (or increased dimorphism in body mass between sexes), variation in autopodial morphology could reflect developmental changes exclusively associated with differences in body mass. The present study tested the feasibility of these evolutionary scenarios, and verified whether selective pressures related to locomotion in different microhabitats could override the prenatal developmental cues imposed on the digit ratio 2D:4D by differences in body size between males and females. We predict that this morphological feature will be correlated with body size both in males and females of iguanian lizards, but size-corrected digit ratios may exhibit ecological associations even in the absence of significant correlations between body size and microhabitat indices. These ideas were examined using lizard species from three families of Iguania (Iguanidae, Polychrotidae, and Tropiduridae), and the evolution of ecological associations of body size and the digit ratio 2D:4D in iguanian lizards was tested considering males and females separately. All lineages of this clade, without exception, are pentadactylus (i.e. with five digits [73]), so in this group variation in autopodium morphology is given mostly by changes in the lengths of homologous structures, rather than by loss or fusion of osteological elements.

## Methods

### (a) Animals and Measurements

The present study was based on measurements of body size (SVL) and digit lengths (digits II and IV from the front and hind limbs) obtained for 25 species from 3 major families of Iguania: Iguanidae, Polychrotidae, and Tropiduridae (Table 1). Whenever it was possible, we preferred to use specimens from a single population. Up to 20 specimens of each species (see Table 1), including males and females, were measured using a digital caliper (accurate to 0.01 mm). For each species, specimens measured were the largest ones available at the Brazilian Herpetological Collections visited (MZUSP and INPA). Our study was performed using specimens from institutional herpetological collections of two Brazilian Museums of Natural History and, therefore, there was no need of approval from the ethics committee, as the study did not involve capture or manipulation of live animals.

**Table 1:** Means and standard errors (in mm) of SVL and lengths of the digit II and IV; digit lengths are presented for front and hind limbs.

					front limb				hind limb			
	N		SVL		males		females		males		females	
species	males	females	males	females	dig II	dig IV	dig II	dig IV	dig II	dig IV	dig II	dig IV
*U. superciliosus*	9	11	129.71±4.104	123.40±3.560	10.19±0.475	15.30±0.456	10.00±0.517	14.24±0.645	11.90±0.375	27.96±0.609	11.90±0.428	27.47±0.711
*E. divaricatus*	10	11	71.55±2.121	59.40±1.066	5.55±0.104	7.80±0.152	4.50±0.088	6.32±0.159	7.55±0.184	14.67±0.272	5.98±0.147	12.18±0.238
*P. umbra*	9	10	99.55±9.561	76.05±3.654	8.20±0.678	12.82±0.900	6.29±0.167	10.54±0.360	9.59±0.753	22.88±2.374	7.39±0.368	16.55±0.757
*T. spinulosus*	12	6	95.51±3.823	86.77±3.012	8.86±0.340	12.42±0.388	7.65 ±0.173	10.57±0.267	10.09±0.368	17.56±0.507	8.85±0.265	15.71±0.310
*T. hygomi*	9	5	64.78±0.820	47.34±4.440	5.35±0.142	8.09±0.125	4.22±0.392	6.38±0.390	6.96±0.172	15.70±0.235	5.49±0.364	11.80±0.615
*T. itambere*	10	10	70.82±1.120	60.54±1.499	5.60±0.114	7.30±0.197	4.75±0.094	6.46±0.129	6.76±0.128	10.50±0.200	5.79±0.075	9.49±0.170
*T. psammonastes*	9	10	88.58±1.906	62.90±2.999	7.36±0.145	10.00±0.259	5.72±0.245	7.98±0.320	9.59±0.187	18.35±0.360	7.41±0.311	14.15±0.56
*T. cocorobensis*	6	11	64.09±2.443	53.35±2.777	5.47±0.144	7.80±0.127	4.43±0.136	6.28±0.302	7.21±0.100	13.53±0.368	5.50±0.257	11.03±0.340
*T. etheridgei*	10	10	79.71±2.211	68.25±0.743	5.71±0.139	8.01±0.246	4.52±0.110	6.25±0.208	7.55±0.214	13.08±0.278	5.93±0.119	10.66±0.139
*T. montanus*	10	5	82.01±2.652	72.54±5.156	6.66±0.197	9.26±0.253	5.81±1.130	7.95±1.548	7.97±0.242	14.52±0.334	6.80±1.332	11.81±2.477
*T. insulanus*	10	10	90.78±2.195	70.86±1.034	7.42±0.136	9.98±0.193	5.88±0.158	8.09±0.197	8.59±0.298	14.58±0.190	6.85±0.126	11.62±0.193
*T. oreadicus*	10	10	88.64±1.810	76.61±1.973	7.58±0.130	9.90±0.223	6.62±0.146	8.84±0.119	8.98±0.167	15.62±0.284	7.31±0.162	13.12±0.161
*T. hispidus*	8	10	87.04±3.022	78.32±1.741	7.42±0.181	10.13±0.288	6.73±0.100	9.33±0.132	9.37±0.270	15.54±0.363	7.88±0.256	14.20±0.374
*I. iguana*	10	10	196.20±27.191	234.33±27.280	17.12±2.272	23.52±2.673	20.00±2.160	27.48±2.754	20.35±2.194	41.47±4.146	22.40±2.215	48.72±4.989
*S. obesus*	3	4	175.58±14.983	159.05±2.651	11.40±0.572	15.28±1.280	10.45±0.174	12.79±0.174	15.05±0.793	25.08±1.597	11.45±0.463	18.96±0.560
*E. perditus*	10	9	71.41±2.679	68.53±3.337	7.01±0.272	10.36±0.266	7.00±0.360	10.63±0.296	7.93±0.322	19.11±0.624	7.71±0.431	18.54±0.842
*E. iheringii*	10	10	83.39±5.037	90.42±4.891	7.84±0.421	12.01±0.732	8.97±0.627	13.20±0.935	8.25±0.487	21.39±1.185	9.84±0.532	23.12±1.219
*A. transversalis*	4	9	67.99±5.739	66.67±4.972	4.76±0.474	6.80±0.706	4.73±0.384	6.57±0.518	4.70±0.597	12.06±1.219	4.20±0.319	11.32±0.997
*A. punctatus*	10	10	76.80±1.810	70.70±1.794	5.27±0.246	7.41±0.223	4.93±0.152	7.03±0.222	4.76±0.166	12.73±0.362	4.41±0.130	11.98±0.349
*A. olssoni*	10	10	43.46±0.583	38.66±0.470	2.60±0.098	4.14±0.096	2.12±0.126	3.39±0.05	3.54±±0.103	9.01±0.128	2.96±0.090	8.22±0.224
*A. nitens*	10	8	60.81±2.044	58.89±5.079	5.09±0.250	7.43±0.306	5.03±0.818	6.81±1.120	4.60±0.19	12.76±0.486	5.30±0.907	12.42 ±2.060
*A. ortonii*	10	10	51.73±0.626	46.53±0.651	3.22±0.097	5.23±0.192	2.94 ±0.101	4.63±0.131	3.37±0.122	8.44±0.242	3.17±0.120	7.88±0.123
*A. fuscoauratus*	10	10	55.75±4.693	48.30±1.910	3.85±0.506	5.92±0.529	3.04±0.19	5.06±0.192	4.25±0.503	9.86±1.036	3.14±0.217	8.80±0.274
*P. acutirostris*	10	10	108.24±2.458	128.10±1.865	4.95±0.147	7.15±0.213	5.54±0.090	7.78±0.213	5.95±0.130	9.39±0.342	6.47±0.223	9.56±0.205
*P. marmoratus*	10	10	107.45±2.941	121.50±4.040	5.82±0.132	8.70±0.281	6.16±0.202	9.49±0.150	7.21±0.237	12.62±0.379	7.72±0.372	13.96±0.322

**Figure 1:**
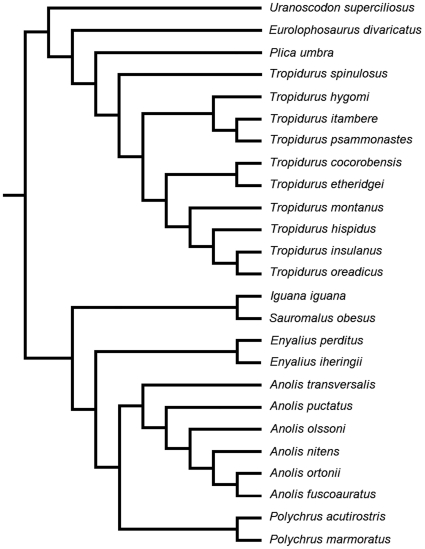
Modified topology of Iguania illustrating the phylogenetic relationships assumed in the present study.

N  =  number of individuals, SVL  =  snout–vent length. Full species' names are listed in Figure 1.

Mean and standard error of SVL and digit ratio 2D:4D were calculated for each species separately for males and females. All morphological traits (i.e. digit lengths and SVL) were log_10_ transformed prior to calculations, and the digit ratio 2D:4D was calculated individually for front and hind limbs, by dividing length of digit II by the length of digit IV (both log transformed), as commonly reported in the current literature [28], [29], [33], [34]. Digit ratio 2D:4D ranged from 0 to 1, where values close to 1 were observed when digits II and IV had very similar lengths and very low values correspond to a morphology where digit IV is much larger than digit II. Digit ratio was significantly correlated with body size both in males and females, as is detailed in the results, and therefore the trait was corrected by SVL. Size-correction was performed following [74], and we computed residuals from a least squares regression analysis performed using a matrix containing information for the expected covariances of the data that are explained by the phylogenetic relationships among taxa. This transformation was performed using the R code described in [74] and a matrix with expected covariances proportional to a topology with Nee's branch lengths (see details for the topology in the item ‘d’ of the present section; diagnostic plots are presented in the supporting information file ‘METHODS- S1’).

We also calculated an index of sexual dimorphism (SDI) in the digit ratio 2D:4D for each species, based on the approach described by [75]. This calculation was performed using the size-corrected 2D:4D digit ratios explained above. Sexual dimorphism indexes are usually calculated by dividing the values of males by those of the females, but [75] suggests that an alternative calculation must be implemented when females exhibit values of the trait that are larger than those observed in the males. Specifically, the idea is to calculate the SDI using the standard approach only when the variable is larger in males: in our study, for all species where the largest values of digit ratio were associated to males SDI was calculated by dividing male's by female's values. According to [75], however, when the trait is larger in females, the SDI shall be calculated as [2-(female/male)]: in our study, this formula was applied for the species where the largest values of digit ratio were observed in the females. The residuals computed from the size-correction analysis detailed above [74] may be either positive or negative, which could influence the signal associated with the calculated SDI because it always results from a division between two traits. Therefore, we have turned all residuals computed from the size-correction transformation into positive values, by adding 1.0, and then calculated SDI as described above. Only right-side values were used in the comparative analyses, as most species studied (87%) were symmetrical between left and right sides.

### (b) Ecological Indices

The evolutionary patterns of digit ratio 2D:4D in males and females of iguanian lizards were analyzed in relation to substrate use. Ecological indices reflecting the percentage of substrate usage were calculated based on published literature (see supporting information file ‘TABLE-S1’), which resulted in five ecological categories: sand, rocks, trunks, perches (branches+leaves), and ground (Table 2). These indices reflect the percentage of lizards from a given population found in each substrate type when performing ecologically relevant activities, as basking, foraging, mating, exploring the territory or evading predators. Values ranged from zero to one: values equal to one for a particular substrate indicate that all individuals of that species were observed on this substrate, while the value ‘zero’ was attributed to ecological categories where no individuals of the species were found. Due to the lack of information about sexual differences in substrate use for most of the species included here, the ecological indices were constructed for the species, and then were used as independent variables in regressions with the morphological variables (male and female digit ratios 2D:4D and SDI, all traits estimated for front and hind limbs).

**Table 2:** Ecological indices (i.e. proportion of individuals observed in a given substrate) estimated from published literature (see supporting information file ‘TABLE- S1’).

	species	sand	rocks	trunks	ground	perches
Tropiduridae	*E. divaricatus*	1.00	0.00	0.00	0.00	0.00
	*P. umbra*	0.00	0.00	0.87	0.05	0.08
	*T. cocorobensis*	1.00	0.00	0.00	0.00	0.00
	*T. etheridgei*	1.00	0.00	0.00	0.00	0.00
	*T. hispidus*	0.00	0.95	0.04	0.01	0.00
	*T. hygomi*	0.90	0.00	0.00	0.00	0.10
	*T. insulanus*	0.00	1.00	0.00	0.00	0.00
	*T. itambere*	0.00	0.99	0.00	0.01	0.00
	*T. montanus*	0.00	0.82	0.05	0.13	0.00
	*T. oreadicus*	0.00	0.35	0.51	0.14	0.00
	*T. psammonastes*	1.00	0.00	0.00	0.00	0.00
	*T. spinulosus*	0.00	0.00	0.97	0.03	0.00
	*U. superciliosus*	0.00	0.06	0.66	0.11	0.17
Polychrotidae	*A. fuscoauratus*	0.00	0.00	0.42	0.16	0.42
	*A. nitens*	0.00	0.00	0.31	0.58	0.11
	*A. olssoni*	0.00	0.00	0.00	0.50	0.50
	*A. ortonii*	0.00	0.35	0.45	0.00	0.20
	*A. punctatus*	0.00	0.00	0.75	0.16	0.09
	*A. transversalis*	0.00	0.00	0.73	0.09	0.18
	*E. iheringi*	0.00	0.00	0.25	0.25	0.50
	*E. perditus*	0.00	0.00	0.50	0.00	0.50
	*P. acutirostris*	0.00	0.00	0.25	0.00	0.75
	*P. marmoratus*	0.00	0.00	0.25	0.05	0.70
Iguanidae						
	*I. iguana*	0.00	0.00	0.35	0.30	0.35
	*S. obesus*	0.00	1.00	0.00	0.00	0.00

Species' names follow Figure 1.

### (c) Statistical Analyses

The presence of sexual dimorphism in the digit ratio 2D:4D in Iguania was first verified by comparing mean values of the ratios between males and females, using paired t-tests (implemented in R; version 2.8.1; R Development Core Team, 2008). Given that body size may have a strong association with the levels of hormones secreted during development, which in turn may also affect the digit ratio 2D:4D, statistical associations between these two morphological traits were inferred using Pearson Correlations implemented in R (version 2.8.1; R Development Core Team, 2008). Correlations were implemented using log transformed variables (i.e. log2D/log4D and logSVL), as detailed above, and were performed using both conventional and phylogenetic statistics. Both analyses were implemented in R; the phylogenetic correlation was performed using independent contrasts, calculated with the packages *geiger* and *picante* and the topology with Nee's branch lengths that is detailed in the following section.

The digit ratios 2D:4D of front and hind limbs were significantly correlated to SVL both in males and females of Iguania (see results), and therefore a size-correction transformation was performed following [74], as detailed above. Morphological data of digit ratios and SDI were then analyzed in relation to substrate usage using both conventional and phylogenetic statistics, and the topology used is detailed in the next section (as well as in the supporting information file ‘METHODS- S1’). These associations between morphological traits (digit ratios in males and females and SDI) and ecological parameters (five categories that reflect the proportion of substrate used by a given species) were performed with REGRESSIONv.2.M for MATLAB (R2008 version for PC). In these analyses, OLS regressions (ordinary least-squares, based on a star phylogeny) and PGLS regressions (phylogenetic generalized least-squares, based on a hierarchical phylogeny) were compared by likelihood ratio tests, which indicate which of the two models better explains the data [13], [76]. The highest likelihood value indicates the model that better fits the data, and a model is considered significantly better than the other only if twice the difference between log-likelihood values is larger than 3.841, which is the critical value for a χ^2^ distribution with 1 d.f. and α = 0.05 [13], [77]. If the difference between log-likelihoods is lower than the critical value, then the phylogenetic model is preferred over the one assuming a star phylogeny.

The statistical design implemented here was based on multiple comparisons, and therefore the critical P values had to be corrected by the number of hypotheses tested. We tested for statistical associations of six traits among five substrates, using two models (star phylogeny and hierarchical topology), which equals to 60 hypotheses. A False Discovery Rate (FDR) analysis was carried out using the QVALUE software package [78] for R (version 2.8.1; R Development Core Team, 2008), with the ‘boostrap’ option. Significant q-values (corresponding to a positive FDR of 5%, [78]) indicate which significant regressions remain ‘true’ after correcting the analyses by the number of hypotheses being tested (in our study, 60).

### (d) Phylogenetic Trees

The use of phylogenetic comparative methods requires the availability of a well-supported topology reflecting the evolutionary relationships among the groups studied. The phylogenetic relationships within Iguania are controversial, and a single hypothesis including all the genera studied here is not available. Because there is more than one phylogenetic hypothesis proposed for some of the genera studied, we have used some criteria for choosing the topology for each taxonomic group: 1) hypotheses with the largest number of the species studied here (in order to minimize the amount of compilations); 2) if more than one phylogenetic hypothesis for a given group fulfilled condition 1, we have used the topology that was proposed using different datasets (e.g., those including molecular and morphological data), or the one that included the largest amount of characters (see criteria used by [79]). We combined the published phylogenetic hypotheses available for several iguanian groups (within families: [80]; Tropiduridae: [81]; Polychrotidae: [82]; see supporting information file ‘METHODS-S1’ for details) into a single topology for the species studied here, using the program TREEVIEW for PC (Fig. 1).

The use of statistical methods based on phylogenies also presumes the adoption of branch lengths in units proportional to the expected variance of character evolution or to divergence time (reviewed in [13], [83]). It is suggested that the clade Iguania may have originated between 150 [84] and 180 Mya [85], but in the present study we have used a topology that is a composite of multiple phylogenetic hypotheses, and therefore precise estimates of phylogenetic branch lengths in units of divergence times or genetic distances are unavailable. Because the published phylogenetic hypotheses available for iguanians do not include the same molecular markers, we cannot run a new analysis to generate branch lengths proportional to rate of nucleotide substitutions. For these reasons, we have tested four different types of arbitrary branch lengths, including all  =  1 (Constant) [86], Pagel [87], and Nee (cited in [88]), using the MS-DOS computer program PDTREE [89]–[91]. Nee branch lengths provided the best standardization of phylogenetically independent contrasts, which was indicated by the absence of statistically significant trends in all diagnostic plots produced using these branch lengths [92]. These diagnostic plots are presented in the supporting information file ‘FIGURE-S1’.

## Results

**Figure 2:**
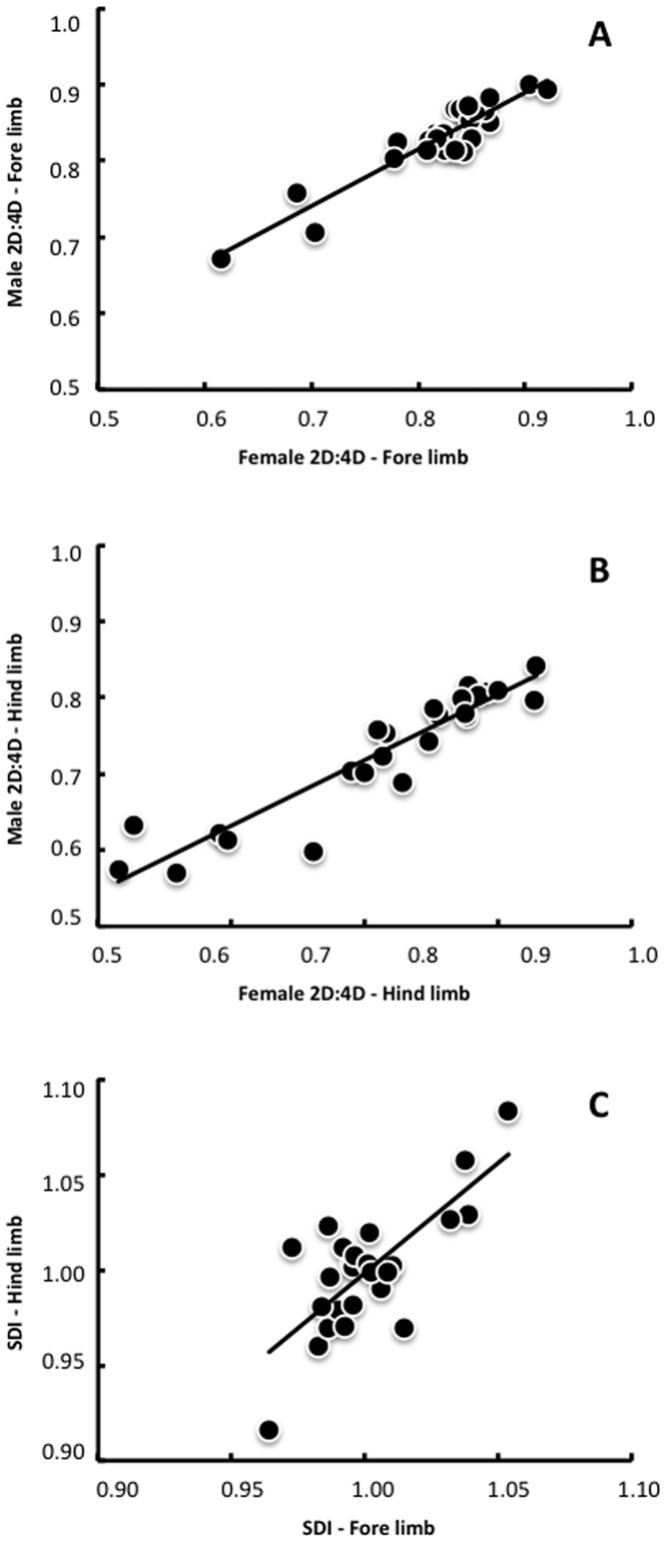
Scatterplots relating the digit ratio 2D:4D of males and females in the front limb (A) and the hind limb (B), and also relating the sexual dimorphism index (SDI) of the front and hind limbs (C). Regression lines are included.

The present study tested the hypothesis that variation in the digit ratio 2D:4D evolved in association with ecological divergence in Iguania, despite the expected effect of body size on the concentrations of steroids that ultimately determine the lengths of digits in the autopodium. In other words, we predict that the selective pressures imposed by locomotion in different microhabitats might overcome a developmental program imposed exclusively by the association of body size with the levels of hormones secreted in the embryo, so that size-corrected digit ratios might exhibit ecological associations even in the absence of significant correlations between body size and microhabitat indices. In the species studied, males overall exhibited larger digit ratios than females, both in the front limbs (Fig. 2A; paired t-tests: t = −2.086, d.f. = 24, P = 0.048) and in the hind limbs (Fig. 2B; paired t-tests: t = −2.496, d.f. = 24, P = 0.020). Differences in digit ratios might be explained by variation in body size, as all correlations between SVL and the digit ratios were significant, both in males and in females (Table 3), and males overall exhibited larger body sizes than females (paired t-tests: t = −3.565, d.f. = 24, P = 0.002). Our main hypothesis, however, predicts that selective pressures imposed by locomotion in different microhabitats might overcome part of the developmental program imposed by the association of body size with the levels of hormones secreted in the embryo. If this is true, then size-corrected digit ratios would be correlated microhabitat usage even when body size does not exhibit such ecological associations. In fact, SVL was not correlated with ecological indices, but both the digit ratios and the sexual dimorphism indices (SDI) exhibited significant associations with microhabitat usage (Table 4). Specifically, variation in the digit ratio 2D:4D was negatively correlated to the use of perches in Iguania, as females of species that move often on perches exhibit lower digit ratios in their front limbs (Table 4). Interestingly, such patterns were not identified in males, which may indicate the existence of sexual dimorphism in microhabitat use among arboreal iguanians.

**Table 3:** Results of conventional and phylogenetic correlations between the digit ratio 2D:4D and SVL.

			Conventional	Phylogenetic
Males	Front limb	Coefficient	0.738	0.698
		P	**<0.001**	**<0.001**
	Hind limb	Coefficient	0.670	0.657
		P	**<0.001**	**<0.001**
Females	Front limb	Coefficient	0.712	0.657
		P	**<0.001**	**<0.001**
	Hind limb	Coefficient	0.677	0.712
		P	**<0.001**	**<0.001**

Pearson correlations: all correlations were statistically significant, as indicated by probabilities (P) lower than 0.001. Phylogenetic correlations performed with independent contrasts calculated using Nee branch lengths.

**Table 4:** Results of conventional and phylogenetic regressions between morphological traits and ecological categories.

			CONVENTIONAL					PHYLOGENETIC (Nee branch lengths)				
			sand	rocks	trunks	ground	perches	sand	rocks	trunks	ground	perches
SVL	Males	coefficient	−0.069	0.071	0.054	−0.193	−0.002	−0.036	−0.011	0.080	−0.014	0.011
		P	0.376	0.217	0.591	0.331	0.990	0.565	0.827	0.295	0.918	0.935
		Q	0.376	0.284	0.459	0.355	0.557	0.459	0.517	0.340	0.539	0.539
		likelihood	13.069	13.064	12.794	13.159	12.633	19.423	19.266	19.849	19.245	19.243
SVL	Females	coefficient	−0.144	0.039	0.093	−0.075	0.173	−0.092	−0.024	0.112	0.095	0.135
		P	0.117	0.687	0.436	0.754	0.266	0.227	0.707	0.226	0.563	0.400
		Q	0.218	0.482	0.413	0.492	0.315	0.284	0.482	0.284	0.459	0.391
		likelihood	9.388	8.111	8.357	8.075	8.708	14.941	14.208	14.942	14.315	14.522
2D:4D males	FL	coefficient	0.026	0.029	−0.016	−0.059	−0.096	−0.008	0.008	0.015	0.012	−0.073
		P	0.164	0.124	0.509	0.209	**0.001**	0.670	0.585	0.495	0.761	0.039
		Q	0.264	0.218	0.440	0.284	0.011	0.482	0.459	0.437	0.492	0.110
		likelihood	49.382	49.620	48.546	49.179	54.886	50.946	51.011	51.104	50.897	53.208
	HL	coefficient	0.053	0.075	−0.093	−0.221	−0.091	−0.018	0.043	−0.048	−0.076	0.014
		P	0.089	0.018	0.017	**0.004**	0.082	0.453	0.023	0.097	0.131	0.786
		Q	0.200	0.080	0.080	0.036	0.194	0.416	0.080	0.208	0.218	0.498
		likelihood	36.310	37.824	37.857	39.423	36.380	43.302	45.849	44.513	44.253	43.029
2D:4D females	FL	coefficient	0.039	0.039	−0.021	−0.077	−0.141	0.008	0.023	0.003	−0.016	−0.180
		P	0.108	0.117	0.495	0.219	**<0.001**	0.743	0.257	0.932	0.765	**<0.001**
		Q	0.218	0.218	0.437	0.284	0.000	0.492	0.313	0.539	0.492	0.000
		likelihood	42.839	42.774	41.663	42.243	50.426	42.145	42.799	42.090	42.135	51.054
	HL	coefficient	0.064	0.076	−0.095	−0.204	−0.127	−0.003	0.050	−0.061	−0.055	−0.083
		P	0.059	0.028	0.025	0.016	0.023	0.911	0.013	0.049	0.323	0.126
		Q	0.148	0.084	0.080	0.080	0.080	0.539	0.080	0.130	0.355	0.218
		likelihood	34.577	35.271	35.374	35.813	35.465	40.912	44.294	43.049	41.448	42.205
SDI	FL	coefficient	−0.014	−0.011	0.005	0.022	0.048	−0.016	−0.017	0.012	0.032	0.112
		P	0.217	0.330	0.705	0.441	**0.005**	0.353	0.219	0.574	0.364	**<0.001**
		Q	0.284	0.355	0.482	0.413	0.038	0.369	0.284	0.459	0.372	0.000
		likelihood	62.292	61.972	61.525	61.775	65.878	52.601	52.960	52.298	52.580	59.458
	HL	coefficient	−0.011	−0.002	0.003	−0.018	0.039	−0.015	−0.009	0.014	−0.023	0.108
		P	0.519	0.919	0.899	0.681	0.170	0.981	0.630	0.640	0.660	0.024
		Q	0.441	0.539	0.539	0.482	0.264	0.557	0.480	0.480	0.482	0.441
		likelihood	50.766	50.541	50.544	50.629	51.580	43.493	43.412	43.404	43.391	46.127

Significant regressions classified as ‘true’ by the FDR analysis (Q<0.05) are indicated in bold. FL = front limb, HL = hind limb, SDI = sexual dimorphism index.

The differences in ecological associations of the digit ratio 2D:4D between males and females of Iguania are consistent with the results obtained for the sexual dimorphism index (SDI, size-corrected as described in the methods section). Calculated values of the SDI were statistically equivalent between front and hind limbs (Fig. 2C; paired t-tests: t = 0.321, d.f. = 24, P = 0.751), but this trait was significantly correlated to microhabitat usage only in the front limbs, where increased differences between males and females in digit ratios (given by larger values of SDI) were associated with the use of perches (Table 4).

## Discussion

The presence of sexual dimorphism in the digit ratio 2D:4D has been suggested in representatives of most tetrapod groups (e.g. lizards: [34], [35]; birds: [32]; mammals: [28], [31], [42], [45]–[48]), but to our knowledge this is one of the first studies that uses a comparative framework to explore whether ecological divergence could trigger variation in autopodial morphology that might override the effects imposed on this morphological trait exclusively by sexual dimorphism of body size. Overall, in the species studied here the digit ratio 2D:4D differs between males and females, but the ecological associations identified were restricted to front limbs of females from iguanian lizards that often use perches. Body size was not related to microhabitat usage in the species of Iguania studied, which suggests that the selective pressures imposed by moving on arboreal environments likely modified a developmental program imposed on digit ratios exclusively by variation in hormone levels due to differences in body size. Interestingly, such ecological associations have evolved differently among males and females of iguanian lizards, and larger sexual dimorphism in the digit ratio 2D:4D is observed in the front limbs of arboreal species. We recognize that there may be multiple selecting forces acting on this scenario, so that effects of selection acting for example in male secondary sexual traits, which would be unrelated to habitat usage, might indirectly drive changes in androgens concentrations and affect the 2D:4D digit ratio. However, the patterns identified here suggest a clear association of digit ratios with ecological divergence that is independent of body size in Iguania, although the adaptive significance of the evolution of an autopodium sexually dimorphic in arboreal species remains to be tested.

The evolution of morphological differences between males and females in a given structure, as the autopodium, can be strongly associated with the performance exhibited in specific ecological settings, and very likely occurs in a scenario where the selective pressures acting over the trait are at least slightly distinct among sexes. Along these lines, our data suggests that some species of Iguania may be sexually dimorphic in relation to microhabitat usage, and maybe females of arboreal species differ from males in the diameter of the perches used or the time they spend basking or moving along different surfaces. Unfortunately, ecological information about sexual dimorphism of microhabitat usage in Iguania is still very scarce and limited to few species, so additional data on ecology is necessary to formally test this hypothesis. For example, although microhabitat use is apparently equivalent between males and females of some Tropidurinae species (e.g. *T. itambere* [93]), in this family there are also species that seem sexually dimorphic for ecological patterns (e.g. *E. divaricatus*, Zampieri & Kohlsdorf, pers. obs.; *T. hygomi* [94]). In Polychrotidae, there are some species where males and females apparently differ in microhabitat usage, as *E. iheringi* and *E. perditus* [95]. Given the fragmentation of this information set, our results emphasize the necessity of new data available from future studies focusing on sexual dimorphism and behavioral ecology of tropical lizards.

Evolutionary changes in the digit ratio 2D:4D associated with the use of different microhabitats are particularly interesting in a scenario where males and females exhibit different morphological patterns (as observed in Iguania) because some developmental mechanisms eliciting sexual dimorphism in digit lengths have already been proposed for tetrapods [36], [37], [42]. Specifically, *Hox* gene expression, which coordinates autopodium development, is influenced by sexual steroids [40], [42], and estrogen concentrations affect positively the lengths of digit II while digit IV length is affected by testosterone levels [28, but see 96]. In *Anolis carolinensis* lizards, estrogen concentration remains relatively constant during embryo development, but the testosterone levels reach their maximum concentrations in two developmental stages, the first peak derived from maternal supply around eight days after fecundation, and the second one produced by the embryo at the 24^th^ day of incubation [97]. Digit patterning and elongation starts around the 11^th^ day of incubation, so this process could be directly affected by maternal-origin testosterone [32], [33]. Moreover, as the process of digit growth may last until the second peak of testosterone (for details about variation in hormone concentrations during development, see [97], [98]), it is possible that digit length is also influenced by the amount of hormones produced by the embryo [99].

Developmental mechanisms may also explain how the ecological associations of the digit ratio 2D:4D observed in females of arboreal iguanians are restricted to the front limbs. Hind limb development often ends before the complete formation of the front limbs [100], and therefore changes in the timing and concentration of steroids secretion may also affect differently the development of these structures [101]. Although the present study did not quantify changes in steroids secretion during embryo development, it is possible to speculate that these evolutionary changes may have been modulated by modifications in hormone concentrations that differently affected front and hind limbs because development of these structures is temporally detached [101], [102]. A formal test of this hypothesis depends on embryo availability, which is constrained by the unsuitability of reproduction in captivity for most of the species studied.

The evolution of different morphological patterns of digit ratio in association with changes in microhabitat use possibly has biomechanical implications for locomotion on different surfaces, besides the likely effects on other ecological activities, as basking, foraging and mating. Grasping ability is particularly relevant for arboreal species [53], [59], [62], [103], where the front limb plays a major role of keeping the center of mass close to the surface [11], [62], and evolutionary changes in foot morphology associated to the use of branches (and rocks) have been reported in Tropidurinae lizards [13]. Females of iguanian species that often use perches tend to exhibit smaller digit ratios 2D:4D (i.e., digit IV is much longer than digit II); this may reflect sexual dimorphism in microhabitat usage in species from forested environments, although these data are not available from the current ecological literature on tropical lizards. Arboreal primates, for example, exhibit the fourth digit considerably longer than the second one [104], and lizards with longer digits presumably reach perches far apart easier and have enough body weight support when travelling among branches [103]–[105].

The adaptive meaning of the evolutionary patterns identified in Iguania for the digit ratio 2D:4D of males and females will certainly be clarified when performance data of males and females running on different substrates, in addition to ecological information of sexual dimorphism in microhabitat usage, are incorporated to the present framework. To our knowledge, studies based on phylogenetic methods that compare the evolution of digit ratio 2D:4D between males and females in a scenario contrasting ecological divergence with body size effects are rare or inexistent. In this context, our study provides evidence that ecological associations of the digit ratio 2D:4D, which are restricted to front limbs of iguanian females, evolved independently of associations between body size and microhabitat usage. Therefore, selective pressures related to ecological divergence may accommodate changes in steroids concentrations during embryo development that override the developmental program imposed exclusively by variation in body size. These results, together with the subsequent discussion, provide substrate for further investigation on developmental mechanisms associated to the evolution of adaptive changes occurring during the colonization of novel environments by squamates.

## Supporting Information

Figure S1 **Diagnostic plots using Nee branch lengths.**
(TIFF)

Methods S1 **Description of the phylogenetic trees used in the present study.**
(DOC)

Table S1 **Information about the literature source used to calculate the ecological indices used in the regressions with morphological traits of digit ratio 2D:4D.**
(DOCX)
