# Supplementary figures and images for: Evolution of Sexual Dimorphism in the Digit Ratio 2D:4D - Relationships with Body Size and Microhabitat Use in Iguanian Lizards
Source: PLoS One. 2011 Dec 5;6(12):e28465. doi: 10.1371/journal.pone.0028465 (PMC3230595; doi:10.1371/journal.pone.0028465)

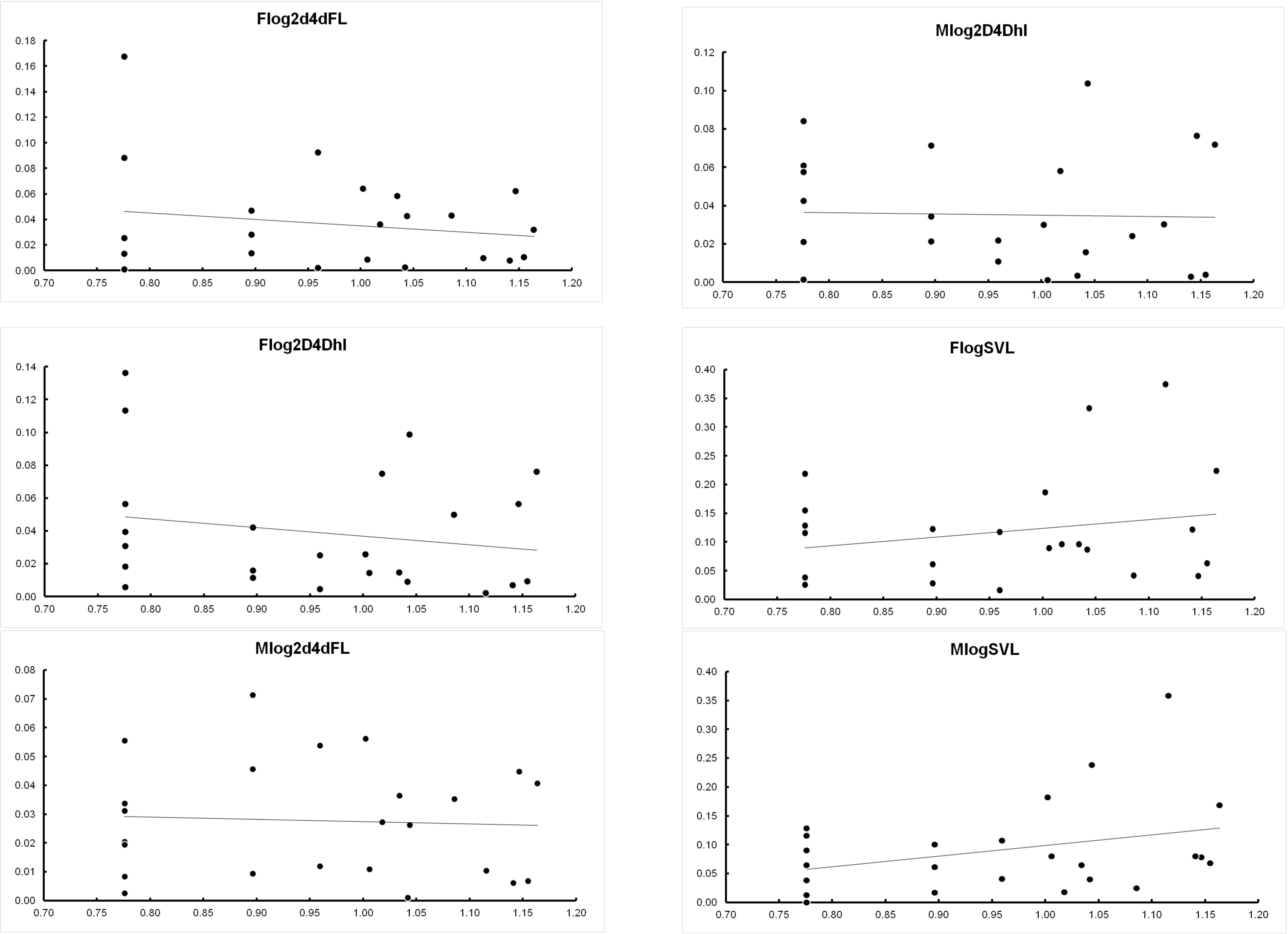

Supplement: Figure S1 — Diagnostic plots using Nee branch lengths. (TIFF) [file pone.0028465.s001.tiff]
