# Supplementary material for: Evolution of Sexual Dimorphism in the Digit Ratio 2D:4D - Relationships with Body Size and Microhabitat Use in Iguanian Lizards
Source: PLoS One. 2011 Dec 5;6(12):e28465. doi: 10.1371/journal.pone.0028465 (PMC3230595; doi:10.1371/journal.pone.0028465)
Supplement: Methods S1 — Description of the phylogenetic trees used in the present study. (DOC) [file pone.0028465.s002.doc]

SUPPLEMENTARY INFORMATION

S1-METHODS

1. *Phylogenetic trees*

The phylogenetic relationships between the families Iguanidae, Tropiduridae and Polychrotidae (Fig. 1) were estimated from the hypothesis proposed by Conrad *et al.* (2007), which presents the largest taxa number and several morphological characters and paleontological information. Within Iguanidae, two species were included in the present study and, as the family is considered monophyletic (De Queiroz, 1987; Etheridge & De Queiroz, 1988; Frost & Etheridge, 1989; Schulte *et al*., 2003), the two genera studied here were treated as sister-groups. The phylogenetic relationships within Tropiduridae were based on the hypothesis of Frost *et al.* (2001a), which is based on morphological and molecular characters, and includes the largest number of taxa studied here. The relationships within Polychrotidae were based on Frost *et al.* (2001b), which combined morphological and molecular data. Because the present study included only two species of *Enyalius* and two species of *Polychrus*, each pair was treated as sister-species in our combined topology. The relationships among *Anolis* were based on the hypothesis of Poe (2004), which contains all the species included here and is congruent with previous studies (Williams, 1976a, b; Savage & Guyer, 1989; Bumell & Hedges, 1990).

LITERATURE CITED

Conrad, J. L., Rieppel, O., Grande, L. 2007. A Green river (eocene) polychrotid (Squamata: Reptilia) and a re-examination of Iguanian systematic. *J. Paleontol*. **81**, 1365–1373.

De Queiroz, K. 1987. Phylogenetic systematics of iguanine lizards: A comparative osteological study. *Univ. California Publ. Zool*. **118**, 1-203.

Etheridge, R., De Queiroz, K. 1988. A phylogeny of Iguanidae. In: *Phylogenetic relationships of the lizards families: Essays commemorating Charles L. Camp*. (eds. Estes, R. & Pregil, G.K.), 268-283. Stanford, CA: Stanford University Press.

Frost, D. R., Etheridge, R. 1989. A phylogenetic analysis and taxonomy of iguanian lizards (Reptilia: Squamata). *Miscell. Publs.*, **81**, 1–65.

Schulte, J. A.,II, Valladares, J. P., Larson, A. 2003. Phylogenetic relationships within Iguanidae inferred using molecular and morphological data and a phylogenetic taxonomy of iguanian lizards. *Herpetologica* **59**, 399–419.

Frost, D. R., Rodrigues, M. T., Grant, T. 2001a. Phylogenetics of the lizard genus *Tropidurus* (Squamata: Tropiduridae: Tropidurinae): direct optimization, descriptive efficiency, and sensitivity analysis of congruence between molecular data and morphology. *Mol. Phyl. Evol*. **21**, 352–371.

Frost, D. R., Etheridge, R., Janies, D., Titus, T. A. 2001b. Total evidence, sequence alignment, evolution of Polychrotid lizards, and a reclassification of the *Iguania* (Squamata: Iguania). *Am. Mus. Novit.* **3343**, 1–38.

Poe, S. 2004. Phylogeny of anoles. *Herp. Monog.* **18**, 37–89.

Williams, E. E. 1976a. West Indian anoles: a taxonomic and evolutionary summary 1. Introduction and a species list. *Breviora* **440**, 1–21.

Williams, E. E. 1976b. South American anoles: the species groups. *Pap. Av. Zool*. (São Paulo) **29**, 259–268.

Savage, J. M., Guyer, C. 1989. Infrageneric classification and species composition of the anole genera, *Anolis, Ctenonotus, Dactyloa, Norops*, and *Semiurus* (Sauria: Iguanidae). *Amphibia-Reptilia* **10**, 105–116.

Burnell, K. L., Hedges S. B. 1990. Relationships of West Indian *Anolis* (Sauria: Iguanidae): an approach using slow evolving protein loci. *Carib. J. Sci.* **26**, 7–30.
