# Supplementary material for: Evolution of Sexual Dimorphism in the Digit Ratio 2D:4D - Relationships with Body Size and Microhabitat Use in Iguanian Lizards
Source: PLoS One. 2011 Dec 5;6(12):e28465. doi: 10.1371/journal.pone.0028465 (PMC3230595; doi:10.1371/journal.pone.0028465)
Supplement: Table S1 — Information about the literature source used to calculate the ecological indices used in the regressions with morphological traits of digit ratio 2D:4D. (DOCX) [file pone.0028465.s003.docx]

(TABLE S1) Information about the literature source used to calculate the ecological indices used in the regressions with morphological traits of digit ratio 2D:4D.

|  | species | literature source |
| --- | --- | --- |
| Tropiduridae | *E. divaricatus* | Rodrigues, M. T. 1986. *Pap. Av. Zool.* (São Paulo) **36**, 171–179. |
|  | *P. umbra* | Vitt, L. J. et al. 1997. *Can. J. Zool.* **75**, 1876–1882. |
|  | *T. cocorobensis* | Rodrigues, M. T. et al. 1989. *Rev. Bras. Gen.* **12**, 747–759; Rodrigues, M. T. 1996. *J. Herp.* **30**, 513–523. |
|  | *T. etheridgei* | Vitt, L. J. 1991. *J. Herp.* **25**, 79–90 |
|  | *T. hispidus* | Rodrigues, M. T. 1988. Academia Brasileira de Ciências, Rio de Janeiro, 305–315; Vitt, L. J. 1995. *Occ. Pap. Oklahoma Mus. Nat. Hist*. **1**, 1–29; Vitt, L. J., Zani, P. A. 1996. *Can. J. Zool.* **74**, 1313–1335; Van Sluys, M. et al. 2004. *J. Herp.* **38**, 606–611. |
|  | *T. hygomi* | Vanzolini, P. E., Gomes, N. 1979. *Pap. Av. Zool*. (São Paulo) **32**, 243–259 |
|  | *T. insulanus* | Rodrigues, M. T. 1987. *Arq. Zool*. **31**, 105–230 |
|  | *T. itambere* | Van Sluys, M. 1992. *Rev. Bras. Biol*. **52**, 181–185; Van Sluys, M. 1993. *J. Herp.* **27**, 347–351; Van Sluys, M. 1998. *J. Herp.* **32**, 359–365 |
|  | *T. montanus* | Van Sluys, M. et al. 2004. *J. Herp.* **38**, 606–611; Rodrigues, M. T. 1987. *Arq. Zool*. **31**, 105–230; Kiefer, M. C., Sazima, I. 2002. *Cannib.* *Herpetol*. *Rev*. **33**, 36 |
|  | *T. oreadicus* | Colli, G. R. et al. 1992. *J. Herp.* **26**, 66–69; Vitt, L. J., Caldwell, J. P. 1993. *J. Herp.* **27**, 46–52 |
|  | *T. psammonastes* | Rodrigues, M. T. 1988. Academia Brasileira de Ciências, Rio de Janeiro. 305–315; Rodrigues, M. T. 1996. *J. Herp.* **30**, 513–523 |
|  | *T. spinulosus* | Colli, G. R. et al. 1992. *J. Herp.* **26**, 66–69; Vitt, L. J. 1991. *J. Herp.* **25**, 79–90 |
|  | *U. superciliosus* | Howland, J. M. et al. 1990. *Can. J. Zool.* **68**, 1366–1373. |
|  |  |  |
| Polychrotidae | *A. fuscoauratus* | Vitt, L. J. et al. 1996. *J. Trop. Ecol*. **12**, 81–101; Vitt, L. J. et al. 2003. *Can. J. Zool.* **81**, 142–156 |
|  | *A. nitens* | Vitt, L. J., Zani, P. A. 1996. *Can. J. Zool.* **74**, 1313–1335; Vitt, L. J. et al. 2001. Copeia, 401–412; Vitt, L. J. et al. 2008. Copeia, 142–151. |
|  | *A. olssoni* | Hertz, P. E. 1979. *J. Herp.* **13**, 329-333 |
|  | *A. ortonii* | Vitt, L. J., Zani, P. A. 1996. *Can. J. Zool.* **74**, 1313–1335. |
|  | *A. punctatus* | Vitt, L. J. et al. 2003. *J. Herp.* **37**, 276–285. |
|  | *A. transversalis* | Vitt, L. J. et al. 2003. *J. Herp.* **37**, 276–285. |
|  | *E. iheringi* | Liou, N. S. 2008. Universidade de São Paulo, Instituto de Biociências, Departamento de Zoologia, São Paulo |
|  | *E. perditus* | Liou, N. S. 2008. Universidade de São Paulo, Instituto de Biociências, Departamento de Zoologia, São Paulo; Sousa, B. M., Cruz, C. A. G. 2008. *Iheringia Sér. Zool*. **98**, 260-265. |
|  | *P. acutirostris* | Vitt, L. J., Lacher, T. E. Jr. 1981. *Herpetologica* **37**, 53-63; Ávila-Pires, T. C. 1995. Zoologische Verhandelingen Leiden. |
|  | *P. marmoratus* | Ávila-Pires, T. C. 1995. Zoologische Verhandelingen Leiden; Vitt**,** L. J. et al. 2008. Áttema Design Editorial, Manaus |
| Iguanidae |  |  |
|  | *I. iguana* | Swanson, P. L. 1950. *Herpetologica* **6**, 187-193; Hirth, H. F. 1963. *Ecol. Soc. Am.* **44**, 613-615 |
|  | *S. obesus* | Johnson, S. R. 1965. *Am. Midl. Nat*. **73**, 1-29; Nagy, K. A. 1973. *Copeia* **1977**, 93-102 |
|  |  |  |
